# Draft Genome Sequence of *Clostridium scatologenes* ATCC 25775, a Chemolithoautotrophic Acetogenic Bacterium Producing 3-Methylindole and 4-Methylphenol

**DOI:** 10.1128/genomeA.00459-14

**Published:** 2014-05-15

**Authors:** Yoseb Song, Yujin Jeong, Hyeon Seok Shin, Byung-Kwan Cho

**Affiliations:** a Department of Biological Sciences and KAIST Institute for the BioCentury, Korea Advanced Institute of Science and Technology, Daejeon, Republic of Korea; b Intelligent Synthetic Biology Center, Daejeon, Republic of Korea

## Abstract

*Clostridium scatologenes* ATCC 25775 is a strictly anaerobic and chemolithoautotrophic acetogenic bacterium that converts syngas into multi-carbon compounds such as acetate, indole, 3-methylindole, and 4-methylphenol. Here we report the draft genome sequence of *C. scatologenes* ATCC 25775 (7.3 Mbp) to elucidate its metabolic pathway for syngas fermentation.

## GENOME ANNOUNCEMENT

Carbon monoxide (CO), carbon dioxide (CO_2_), and hydrogen (H_2_) are main components in syngas, which are key precursors for the sustainable production of value-added chemicals through microbial metabolism known as syngas fermentation (1, 2). Compared to thermo-chemical processes like Fischer-Tropsch synthesis, the usage of acetogenic bacteria (acetogens) during syngas fermentation has important advantages for the production of various industrial commodities. These include higher catalytic specificity, lower energy costs, greater resistance to catalyst poisoning, and independence of the CO/H_2_ ratio (3). As one of the syngas-utilizing acetogens, *Clostridium scatologenes* ATCC 25775 oxidizes hydrogen to grow chemolithoautotrophically and fix CO_2_ or CO into acetate as the main end product under anaerobic conditions (4). The bacterium also produces malodorous chemicals such as 3-methylindole and 4-methylphenol, which are widely used and intermediates the production of other chemicals such as antioxidants and fixatives (4, 5). Despite the metabolic capabilities of *C. scatologenes* as a chemical producer from syngas, lack of genetic information limits strain engineering to design the production host for the useful chemicals. To understand its physiological and metabolic properties, we obtained the draft genome sequence of the acetogen.

For the isolation of its genomic DNA, *C. scatologenes* were grown under anaerobic conditions using the medium reported previously (6). Genomic DNA was isolated using a Wizard Genomic DNA purification kit (Promega) and fragmented using Covaris S220 (Covaris, Inc.). The Illumina paired-end library was prepared using a TruSeq kit (Illumina, Inc.) and sequenced using a MiSeq v2 instrument with the 250 bp paired-end read recipe. The collected reads were trimmed with the default parameters using CLC Genomics Workbench (CLC bio). A total of 1,118,842,164 bases in 7,455,788 reads were then assembled using Velvet (version 1/2/08) (7), resulting in 174 contigs, the largest of which is 472,553 bp. With the assembled genome, rRNAs and tRNAs were predicted using RNAmmer 1.2 and tRNAscan-SE 1.21, respectively (8, 9) and annotation was performed using Rapid Annotation using Subsystem Technology (10).

The draft genome sequence of *C. scatologenes* is 7,353,834 bases, comprising 6,842 predicted open reading frames, 118 tRNA genes, and 13 rRNA genes. It shows a G+C content of 30.2%, consistent with a previous report (31 mol% by nuclease digest) (11). 16S rRNA gene sequence analysis shows that *C. scatologenes* is closely related to *C. carboxidivorans* (99.9%) and *C. drakei* (99.8%) (11, 12). However, the genome of *C. scatologenes* is larger than that of *C. carboxidivorans* (5.63 Mbp, accession number ACVI01000000) and *C. drakei* (5.64 Mbp, accession number JIBU00000000). As expected, *C. scatologenes* uses the Wood-Ljungdahl pathway for fixing CO_2_ to organic carbon via acetyl-CoA. *C. scatologenes* has tryptophan 2-monooxigenase (EC 1.13.12.3) and aliphatic amidase (EC 3.5.1.4), indicating that it contains a metabolic pathway from tryptophan to 3-methylindole via indoleacetic acid (4). The draft genome sequence of *C. scatologenes* will provide genetic information for the understanding of its metabolic capabilities for syngas fermentation.

### Nucleotide sequence accession number.

The draft genome sequence of *C. scatologenes* has been deposited at the DDBJ/EMBL/GenBank database under the accession no. JIDX00000000. The version described in this paper is the first version.

## References

[1] Schiel-Bengelsdorf B Dürre P 2012 Pathway engineering and synthetic biology using acetogens. FEBS Lett. 586:2191–2198. 10.1016/j.febslet.2012.04.043 22710156

[2] Bruno-Barcena JM Chinn MS Grunden AM 2013 Genome sequence of the autotrophic acetogen *Clostridium autoethanogenum* JA1-1 strain DSM 10061, a producer of ethanol from carbon monoxide. Genome Announc. 1:e00628-13. 10.1128/genomeA.00628-13 23950130 PMC3744686

[3] Bredwell MD Srivastava P Worden RM 1999 Reactor design issues for synthesis-gas fermentations. Biotechnol. Prog. 15:834–844. 10.1021/bp990108m 10514253

[4] Whitehead TR Price NP Drake HL Cotta MA 2008 Catabolic pathway for the production of skatole and indoleacetic acid by the acetogen *Clostridium drakei*, *Clostridium scatologenes*, and swine manure. Appl. Environ. Microbiol. 74:1950–1953. 10.1128/AEM.02458-07 18223109 PMC2268313

[5] Jensen MT Cox RP Jensen BB 1995 3-methylindole (skatole) and indole production by mixed populations of pig fecal bacteria. Appl. Environ. Microbiol. 61:3180–3184 7487051 10.1128/aem.61.8.3180-3184.1995 PMC167595

[6] Kridelbaugh D Doerner KC 2009 Development of a defined medium for *Clostridium scatologenes* ATCC 25775. Lett. Appl. Microbiol. 48:426–432. 10.1111/j.1472-765X.2008.02546.x 19187496

[7] Zerbino DR Birney E 2008 Velvet: algorithms for de novo short read assembly using de Bruijn graphs. Genome Res. 18:821–829. 10.1101/gr.074492.107 18349386 PMC2336801

[8] Lagesen K Hallin P Rødland EA Staerfeldt H-H Rognes T Ussery DW 2007 RNAmmer: consistent and rapid annotation of ribosomal RNA genes. Nucleic Acids Res. 35:3100–3108. 10.1093/nar/gkm160 17452365 PMC1888812

[9] Lowe TM Eddy SR 1997 tRNAscan-SE: a program for improved detection of transfer RNA genes in genomic sequence. Nucleic Acids Res. 25:955–964. 10.1093/nar/25.5.0955 9023104 PMC146525

[10] Aziz RK Bartels D Best AA DeJongh M Disz T Edwards RA Formsma K Gerdes S Glass EM Kubal M Meyer F Olsen GJ Olson R Osterman AL Overbeek RA McNeil LK Paarmann D Paczian T Parrello B Pusch GD Reich C Stevens R Vassieva O Vonstein V Wilke A Zagnitko O 2008 The RAST server: Rapid Annotations using Subsystems Technology. BMC Genomics 9:75. 10.1186/1471-2164-9-75 18261238 PMC2265698

[11] Liou JS Balkwill DL Drake GR Tanner RS 2005 *Clostridium carboxidivorans* sp. nov., a solvent-producing clostridium isolated from an agricultural settling lagoon, and reclassification of the acetogen *Clostridium scatologenes* strain SL1 as *Clostridium drakei* sp. nov. Int. J. Syst. Evol. Microbiol. 55:2085–2091. 10.1099/ijs.0.63482-0 16166714

[12] Bruant G Lévesque MJ Peter C Guiot SR Masson L 2010 Genomic analysis of carbon monoxide utilization and butanol production by *Clostridium carboxidivorans* strain P7. PLOS ONE 5:e13033. 10.1371/journal.pone.0013033 20885952 PMC2946384

